# Redeployment of automated *MrBUMP* search-model identification for map fitting in cryo-EM

**DOI:** 10.1107/S2059798321009165

**Published:** 2021-10-20

**Authors:** Adam J. Simpkin, Martyn D. Winn, Daniel J. Rigden, Ronan M. Keegan

**Affiliations:** a Institute of Structural, Molecular and Integrative Biology, University of Liverpool, Liverpool L69 7ZB, United Kingdom; b UKRI–STFC, Rutherford Appleton Laboratory, Research Complex at Harwell, Didcot OX11 0FA, United Kingdom

**Keywords:** *MrBUMP*, molecular replacement, cryo-EM, GroEL

## Abstract

In crystallography, the phase problem can often be addressed by the careful preparation of molecular-replacement search models. This has led to the development of pipelines such as *MrBUMP* that can automatically identify homologous proteins from an input sequence and edit them to focus on the areas that are most conserved. Many of these approaches can be applied directly to cryo-EM to help discover, prepare and correctly place models (here called cryo-EM search models) into electrostatic potential maps. This can significantly reduce the amount of manual model building that is required for structure determination. Here, *MrBUMP* is repurposed to fit automatically obtained PDB-derived chains and domains into cryo-EM maps. *MrBUMP* was successfully able to identify and place cryo-EM search models across a range of resolutions. Methods such as map segmentation are also explored as potential routes to improved performance. Map segmentation was also found to improve the effectiveness of the pipeline for higher resolution (<8 Å) data sets.

## Abstract 2

The modification of a crystallographic molecular-replacement pipeline for use in fitting search models to cryo-EM maps is described.

## 1. Introduction   

Cryogenic electron microscopy (cryo-EM) has rapidly become one of the main experimental methods for determining macromolecular structures, alongside macromolecular X-ray crystallography (MX) and nuclear magnetic resonance (NMR) (Nicholls *et al.*, 2018 ▸). Whilst at present the vast majority of structures deposited in the Protein Data Bank (PDB; Berman *et al.*, 2000 ▸) have been determined by MX (>145 000) and NMR (>13 000), cryo-EM (>5000) is rapidly increasing in popularity. This has been, in part, due to recent advances in instrumentation and software that have resulted in a ‘resolution revolution’ (Faruqi & McMullan, 2011 ▸; Lyumkis *et al.*, 2013 ▸; Kühlbrandt, 2014 ▸; Scheres, 2014 ▸).

Cryo-EM reconstructions cover a large range of resolutions and the resolution determines how the maps are modelled. Indeed, the resolution may vary within a single reconstruction, implying different modelling approaches for different regions. When higher resolution (<4 Å) data are available, it is possible to perform *de novo* model building using software such as *Buccaneer* (Hoh *et al.*, 2020 ▸), *ARP*/*wARP* (Chojnowski *et al.*, 2021 ▸), *phenix.trace_and_build* (Terwilliger *et al.*, 2020 ▸) and *RosettaES* (Frenz *et al.*, 2017 ▸). At lower resolutions, prior information is typically required in the form of an existing atomic model. These models can then be fitted into the map using programs such as *DockEM* (Roseman, 2000 ▸), *MDFF* (Trabuco *et al.*, 2008 ▸), *MOLREP* (Vagin & Teplyakov, 2010 ▸), *CHOYCE* (Rawi *et al.*, 2010 ▸), *DireX* (Wang & Schröder, 2012 ▸), *Flex-EM* (Joseph *et al.*, 2016 ▸), *Rosetta* (Wang *et al.*, 2016 ▸), *phenix.map_to_model* (Terwilliger *et al.*, 2020 ▸) and *cryo_fit* (Kim *et al.*, 2019 ▸).

*MrBUMP* was originally developed as a pipeline that sought to automate protein crystal structure phasing through molecular replacement (MR) (Keegan *et al.*, 2018 ▸; Winn & Keegan, 2007 ▸). *MrBUMP* has been developed to use state-of-the-art bioinformatic programs such as *phmmer* (Eddy, 2011 ▸) and *HHpred* (Söding *et al.*, 2005 ▸; Zimmermann *et al.*, 2018 ▸) to identify even distant homologues for a given sequence. These homologues are then automatically prepared as MR search models for use in MR applications such as *Phaser* (McCoy *et al.*, 2007 ▸) and *MOLREP*. In MR, testing a large number of models can be paramount for solving the phase problem. In cryo-EM, the selection of an initial model for refinement into a cryo-EM map can be somewhat arbitrary and/or rely exclusively on sequence identity. Using a systematic and quantitative approach, such as *MrBUMP*, can solve this problem by screening a large number of models and identifying the one which best fits into the map according to some chosen criterion.

Here, we explore the use of *MrBUMP* to identify cryo-EM search models and place them in cryo-EM maps. GroEL data sets covering a range of resolutions (3.26–18 Å) were used to assess *MrBUMP*. We find that *MrBUMP* is successfully able to identify suitable cryo-EM search models and is able to place them into maps with resolutions as low as 18 Å. Additionally, we find that map segmentation can improve the performance of *MrBUMP* for higher resolution data sets (<8 Å) whilst also reducing the run time.

## 2. Methods   

### 2.1. Data-set selection   

GroEL was selected as an exemplar system since the EMDB (Abbott *et al.*, 2018 ▸) contains a large number of GroEL maps that cover a wide range of resolutions and the PDB contains a large number of GroEL homologues covering a range of sequence identities (24.9–100%). In addition, GroEL comprises three domains which undergo a conformational change in the presence of the ‘lid-like’ co-chaperone protein GroES in a cycle driven by ATP hydrolysis. The GroEL complex can therefore be considered to adopt either an open or a closed state. For this study, 12 data sets from the EMDB were selected as target maps (Table 1 ▸). These differed in resolution (3.26–18 Å), but were from the same source organism (*Escherichia coli*), were in the same conformation (closed) and lacked GroES.

### 2.2. Map segmentation   

We trialled the *MrBUMP* pipeline against maps of the full GroEL complex and against maps of a single monomer. Segmented maps were generated for the GroEL data sets using *Segger* from *UCSF Chimera* (version 1.5; Pettersen *et al.*, 2004 ▸), where repeated rounds of automated smoothing/grouping were performed until there were 14 segments corresponding to the 14 molecules of the structure. 11 of our 12 data sets had *C*7/*D*7 symmetry imposed during reconstruction, and therefore the segments produced were very similar. For EMDB entry EMD-5143, where no symmetry was applied, the segments are still broadly similar, but it is conceivable that segment selection might have a small impact on map fitting.

### 2.3. Modifications to *MrBUMP*   

*MrBUMP* has been modified so that it can accept cryo-EM maps and perform molecular docking and refinement using *MOLREP* and *REFMAC*, borrowing the approach used in *CCP-EM* of exploiting the spherically averaged phased translation function (SAPTF) option (Vagin & Isupov, 2001 ▸) to fit the cryo-EM search models into the maps. The SAPTF option searches a map by scoring the spherically averaged density of the cryo-EM search model at each grid point in the MR translation search against the spherically averaged density of the target map in a sphere of radius equivalent to that of the sphere generated by the cryo-EM search model around that point. When successful, the placement corresponds to the correct positioning of the centre of mass of the cryo-EM search model. A subsequent local rotation search is used to find the correct orientation of the cryo-EM search model. This method can be advantageous for the placement of distant homologues as well as that of cryo-EM search models constituting only a small part of the overall target structure. Originally designed for fitting MR search models to partially phased X-ray crystallography electron-density maps, it works well for cryo-EM maps, where the phases are known and the maps are clearly defined, in contrast to the partially resolved X-ray maps.

**Figure 1:**
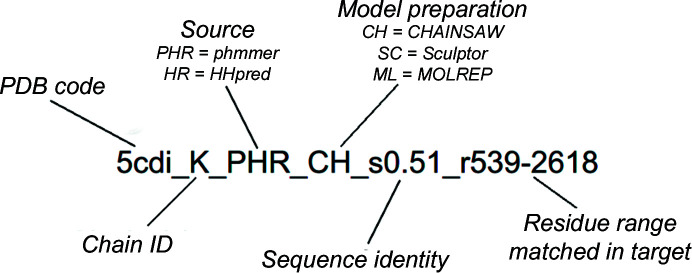
The *MrBUMP* search-model naming convention. Source is the sequence-alignment program used to find the search model based on its similarity to the target.

The modular nature of *MrBUMP* means that alternative molecular-docking and refinement programs may be implemented in future versions. The cryo-EM mode of *MrBUMP* has been made available on the command line as follows: 
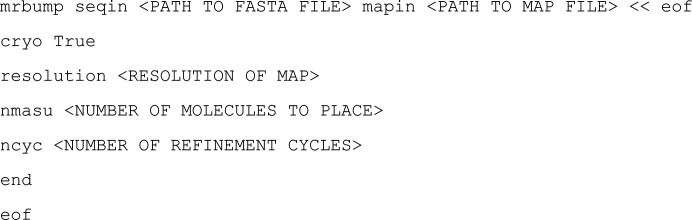


Search-model names in *MrBUMP* contain some details of where the model comes from, how it was prepared and its relation to the target in terms of sequence identity and the residue range in the target that it matches. Fig. 1 ▸ illustrates the details of this convention. ‘Model preparation’ is the application used to generate a ‘mixed’ model, where the original coordinates are modified based on the sequence alignment to the target. This includes the removal of non-aligned loops and the truncation of the side chains of aligned residues that differ back to the C^α^ or C^β^ atoms. In molecular replacement, this helps to remove parts of the MR search model that are likely to differ from the target structure and to eliminate potential noise in the search for correct placement (Schwarzenbacher *et al.*, 2004 ▸). These approaches should be similarly applicable to searching in cryo-EM maps. In this work, all cryo-EM search models were processed in this way using *CHAINSAW* (Stein, 2008 ▸) from the *CCP*4 suite (Winn *et al.*, 2011 ▸). In this work, *MrBUMP* uses the *phmmer* application to perform the search of known PDB structure sequences for matches to our target sequence. To find a broad range of structural matches with varying identity to the target and corresponding structure variation, we used a redundancy-removed database of PDB sequences. *MrBUMP* has several redundancy-level options ranging from the fully redundant set of sequences to a level where anything with 50% identity to a selected sequence is removed from the database. Here, we have used the 95% option, where anything having a greater than 95% identity to a selected sequence is removed.

### 2.4. Scoring placement   

We calculated the lowest chain-to-chain r.m.s.d. between the placed cryo-EM search models and a correctly positioned reference model. In five out of the 12 cases (see Table 1 ▸), a fitted atomic model had been deposited. For the other seven cases no fitted model was available and therefore a fitted model had to be generated. This was performed by fitting two copies of a closed, heptameric *E. coli* GroEL crystal complex (PDB entry 1oel) into the map using *MOLREP* with the SAPTF protocol described above (Vagin & Teplyakov, 2010 ▸). Where *MOLREP* failed to accurately place the structure, *UCSF Chimera* (version 1.5; Pettersen *et al.*, 2004 ▸) was used to manually place PDB entry 1oel (Braig *et al.*, 1995 ▸) in approximately the correct position before using the ‘fit in map’ local optimization tool. PDB entry 1oel has commonly been used as a cryo-EM search model in GroEL map fitting (Joseph *et al.*, 2016 ▸; Stagg *et al.*, 2008 ▸; Clare *et al.*, 2012 ▸; Ludtke *et al.*, 2001 ▸).

**Figure 2:**
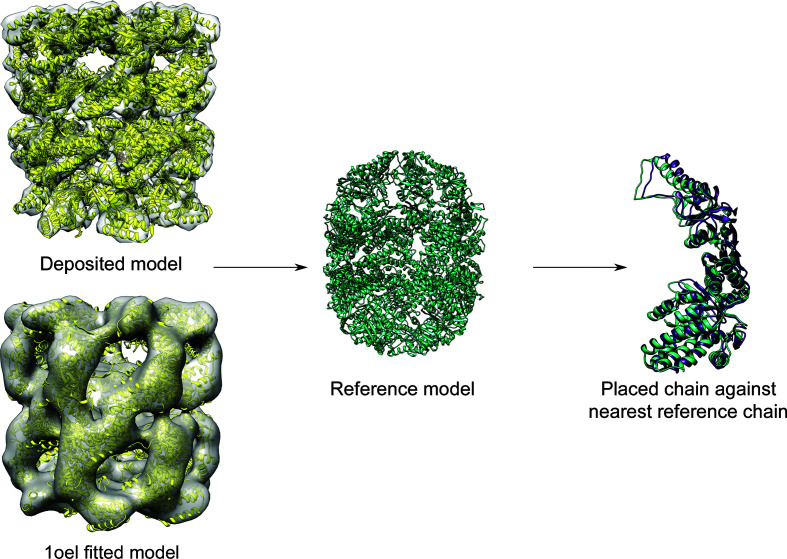
For each data set, the deposited or fitted model (yellow) was used to create a reference model (teal) for each cryo-EM search model by superimposing the cryo-EM search model onto the fitted model with *GESAMT*. This was used to calculate the r.m.s.d. between the reference model and the placed model (purple) on a chain-to-chain basis. Shown here is the deposited model (PDB entry 4aaq) for EMDB entry EMD-1998 (8 Å resolution) and the reference model and the placed cryo-EM search model for PDB entry 1a6d. Also shown is a fitted model (PDB entry 1oel) for EMDB entry EMD-5143 (18 Å); PDB entry 1oel fitted models were used when deposited models were unavailable.

**Figure 3:**
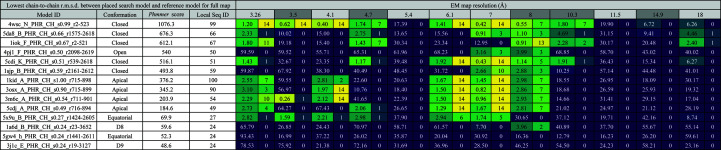
The lowest chain-to-chain r.m.s.d. between the placed cryo-EM search model and a reference model for the full map and the number of molecules that were fitted within 5 Å of the reference model. The columns show 12 target maps at resolutions ranging from 3.26 to 18 Å. A darker shade of grey is used to denote maps where a fitted model was deposited with the data. For the other data sets, fitted models were created using a crystal structure of GroEL (PDB entry 1oel). The rows show the different cryo-EM search models tried, named according to the convention described in Section 2.3.

Each of the 12 fitted models then provided a structure with which to align the cryo-EM search models as a guide to their optimum positioning (Fig. 2 ▸). These aligned cryo-EM search models could then act as a ‘reference model’ against which the solutions could be compared. To generate these reference models, *GESAMT* (Krissinel, 2012 ▸) was used to superimpose the cryo-EM search models onto the fitted model. The r.m.s.d. between each chain in the placed cryo-EM search model and the nearest corresponding chain in the reference model was calculated and the lowest score was reported (Figs. 3 ▸ and 4 ▸). Where more than one cryo-EM search model was placed in the map, we also reported the number of cryo-EM search models that were placed within a 5 Å r.m.s.d. of a reference chain (Fig. 3 ▸).

We also explored the use of the *MOLREP* TFZ score and the *TEMPy* global and local correlation scores (Cragnolini *et al.*, 2021 ▸) to assess the goodness of fit between the placed search models and the map (discussed below).

### 2.5. Computing resources and software versions   

Testing was carried out on a cluster where each node was equipped with twin eight-core Intel Xeon E5-2660 SandyBridge processors running at 2.2 GHz and sharing 64 GB of memory.

The software used in this study corresponds to *CCP*4 version 7.0.068 (Winn *et al.*, 2010 ▸), *MOLREP* version 11.6.04 (Vagin & Teplyakov, 2010 ▸) and *REFMAC* version 5.8.0238 (Murshudov *et al.*, 2011 ▸). The *TEMPy* version corresponds to *CCP-EM* version 1.5.0 (Burnley *et al.*, 2017 ▸). The PDB sequence database used by *MrBUMP* was generated on 10 February 2020.

## 3. Results and discussion   

### 3.1. GroEL case study   

#### 3.1.1. Cryo-EM search-model discovery and characterization   

**Figure 5:**
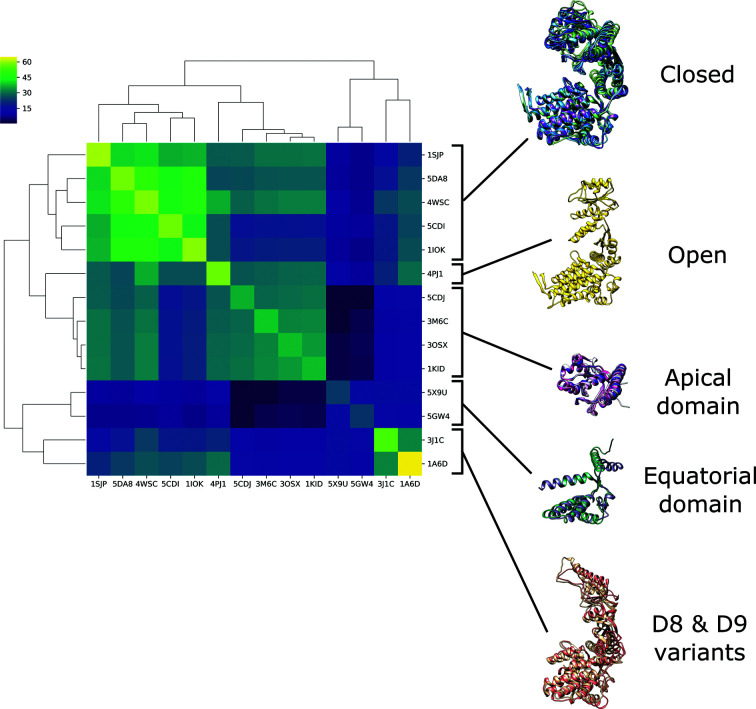
A dendrogram with heatmap showing the results of a *DALI* all-against-all structure comparison. As the colours are based on the *DALI* *Z*-score, they will depend on the size of the model; hence the colour is not consistent on the diagonal. We identified five distinct groups of models: a closed conformation, an open conformation, D8/D9 variants, models relating to the apical domain and models relating to the equatorial domain. The figure was made using *seaborn.clustermap* (Waskom, 2021 ▸) and *UCSF Chimera* (version 1.5).

*MrBUMP* was run using the nonredundant (95%) PDB sequence database as a source of cryo-EM search models matching the sequence of the target. This produced cryo-EM search models across a range of sequence identities. A total of 14 homologues were identified using *phmmer* and these shared between 24% and 100% sequence identity with the *E. coli* sequence (Table 1 ▸). Performing a *DALI* all-against-all structure comparison revealed that there were five distinct groups of cryo-EM search models, which represented the full-length closed conformation, the full-length open conformation, full-length D8/D9 variants, the equatorial domain alone and the apical domain alone (Fig. 5 ▸).

Herein lies a key advantage: through identifying cryo-EM search models in a wide variety of conformations and automating model fitting and refinement, *MrBUMP* has the potential to find the model that best fits the map, even if it has low sequence identity to the target.

#### 3.1.2. Placing cryo-EM search models   

The cryo-EM search models identified by *phmmer* were fitted into the map using *MOLREP* and then put through 20 cycles of refinement with *REFMAC*5 using the modified *MrBUMP* pipeline. Two experiments were run for each of the 12 data sets. The first used *MrBUMP* to place 14 copies of each cryo-EM search model into the full map. The second used *MrBUMP* to place a single copy of the cryo-EM search model into a segmented map (described in Section 2.2). *TEMPy* scoring was initially used to assess how well the placed models fit within the map (Supplementary Table S1). At higher resolutions (<8 Å) the *TEMPy* CC scores were effective at identifying solutions in both full maps (Supplementary Fig. S1) and segmented maps (Supplementary Fig. S2); however, they were ineffective at lower resolutions (≥8 Å). The *MOLREP* TFZ score also provided a good indication of successful placements at higher resolutions, especially for segmented maps (Supplementary Fig. S2). Given that as of 2021 the average single-particle cryo-EM map resolution is 6 Å (https://www.ebi.ac.uk/pdbe/emdb/statistics_sp_res.html/), *MOLREP* and *TEMPy* provide a broadly effective method to validate solutions, but here, in order to assess the accuracy of the placement of the models at all resolution ranges, we used an r.m.s.d. score calculated against a reference model.

#### 3.1.3. Comparing full and segmented maps   

In our first test, *MrBUMP* was used to place 14 copies of each cryo-EM search model into the full EM map. As visualized in Fig. 3 ▸, the high sequence identity (>66%) closed-form homologues (PDB entries 4wsc, 5da8 and 1iok) performed better; that is, each of these models could be placed within 5 Å of the reference model for a large number (42–66%) of the data sets. Conversely, the low sequence-identity (24%) D8/D9-form homologues (PDB entries 1a6d and 3j1c) performed the worst, with models placed within 5 Å of the reference model for only one data set (EMDB entry EMD-6422). The apical domains (PDB entries 1kid, 3osx, 3m6c and 5cdj) could be placed within 5 Å of the reference model for data sets up to 8 Å resolution, beyond which the overall shape of the monomer (both domains) clearly becomes important for accurate map fitting. The apical domains fared better than the equatorial domains; for example, 14 copies of PDB entry 1kid could be placed in the 6.1 Å resolution data set (EMDB entry EMD-5338) compared with only six copies of PDB entry 5x9u. This was to be expected as the apical domains had a higher sequence identity to the target. In addition, the equatorial domains are more closely packed as they form the interface between the two heptamers and therefore small misplacements are more likely to interfere with packing. Interestingly, despite a large variance in sequence identity (49–100%) within the apical domains, they performed nearly identically across the 12 data sets. If we compare the domains with the full models, for example PDB entries 1kid and 4wsc, we can see that despite similar sequence identities, PDB entry 4wsc performs far better across all of the data sets. This highlights the importance of overall shape when fitting models to maps.

The 5.4 Å resolution data set (EMDB entry EMD-1457) appeared to give an anomalous result, with significantly fewer correctly placed models than we might expect. This data set was deposited as part of a study on optimizations for high-resolution single-particle reconstructions (Stagg *et al.*, 2008 ▸). The nominal 5.4 Å resolution was determined using a Fourier shell correlation (FSC) at a cutoff of 0.5. The authors also used *rmeasure* (Sousa & Grigorieff, 2007 ▸) and an FSC_0.5_ calculated against an X-ray crystallographic structure, which gave resolution estimates of 6.9 and 8.1 Å, respectively. In order to assess this, we calculated the *d*_99_. This is the resolution cutoff beyond which Fourier map coefficients are negligibly small. For EMDB entry EMD-1457 the *d*_99_ value comes out at 7.47 Å. This may partly explain why we had difficulties placing the cryo-EM search models within the map, but does not tell the full story as we were able to successfully place models into maps with similar or lower *d*_99_ scores (for example EMDB entry EMD-1997). Given the age of this data set (2008), we surmise that improvements in data collection and image processing may have resulted in success with newer data sets at similar resolutions.

**Figure 6:**
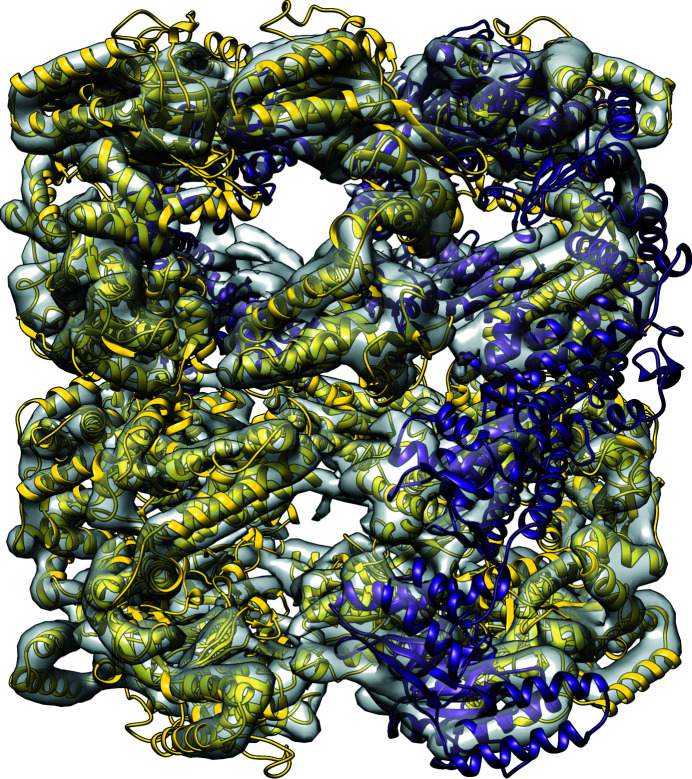
PDB entry 1sjp (59% sequence identity) pre-processed with *CHAINSAW* and fitted into EMDB entry EMD-1997 (7 Å resolution). Correctly placed (according to the r.m.s.d. scoring metric; Section 2.4) chains are shown in yellow and incorrectly placed chains are shown in purple. The incorrectly placed chains correspond to the final four models placed by *MOLREP*. Examining the packing-function scores from *MOLREP* in detail indicated that there were a lot more clashes to deal with when placing these models into the map. Thie figure was made using *UCSF Chimera* (version 1.5).

We observed that the lower sequence identity (50–59%) closed-form homologues struggled with packing in some cases. Fig. 6 ▸ shows the placement of PDB entry 1sjp into EMDB entry EMD-1997, a 7 Å resolution map. The first ten cryo-EM search models were correctly placed within the map; however, the final four models were placed incorrectly due to clashes with the already placed models.

In our second test, *MrBUMP* was used to place a single copy of the cryo-EM search model into a segmented map. Segmenting the maps allows us to focus on the placement of a single cryo-EM search model, thereby avoiding issues with packing. Note, however, that reconstructing the complex through the application of symmetry operations could then result in clashes that would need to be dealt with. If we compare Figs. 3 ▸ and 4 ▸, we can see some general trends. Segmenting the maps significantly improved the placement of cryo-EM search models for higher resolution data sets (<8 Å). Curiously, however, at lower resolutions (≥8 Å) the full unsegmented maps performed better.

An added benefit of using segmented maps was significantly shorter run times (Supplementary Fig. S1). Using segmented maps was more than 14 times faster than the full-map strategy, suggesting that for high-resolution data sets it would be faster and more effective to run 14 segmented map runs than a single full-map run.

### 3.2. SUR1 apo-state case study   

**Figure 7:**
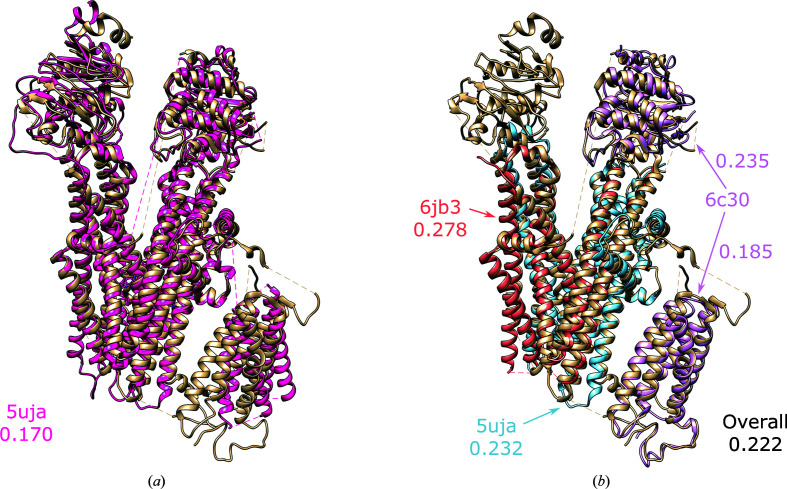
(*a*) Superposition of the nearest homologue (PDB entry 5uja, pink) and the target structure (PDB entry 6pbz, tan). The local *TEMPy* score for this cryo-EM search model is also given. (*b*) Superposition of individual domains (PDB entry 5uja, blue; PDB entry 6jb3, orange; PDB entry 6c3o, light pink) identified and placed by *MrBUMP* and the target structure (PDB entry 6pbz, tan). The local *TEMPy* scores for each of the individual domains is given in addition to an overall score when the domains are considered together.

SUR1 in the apo state (PDB entry 6pzb, 4.55 Å resolution; Martin *et al.*, 2019 ▸) provided a good case study of where the systematic *MrBUMP* approach can help to identify suitable cryo-EM search models when conformational changes make map fitting nontrivial. Here, when searching against a 95% redundancy reduced derivative of the PDB, no homologues were found that adopted the same conformation as the target structure. The closest structure was PDB entry 5uja, a model with only 31% sequence identity to the target (Fig. 7 ▸ *a*) that may have been overlooked if judging suitability based on sequence identity alone. However, even better results were obtained using a domain-based approach exploiting the ability of *MrBUMP* to break cryo-EM search models into domains. In this case, *MrBUMP* was able to place four out of five domains automatically. In its current version, *MrBUMP* looks for a particular number of each domain (one here) and therefore misses the fifth domain (top left in Fig. 7 ▸ *b*), which is homologous to a second domain in the target: the second homologous domain has clearer map features and so cryo-EM search models identified for the fifth domain are placed there in preference. Nevertheless, the domain-based approach leads to a better result with a *TEMPy* local CC score of 0.222 over the four domains, compared with 0.170 for the nearest whole structure in Fig. 7 ▸(*a*).

## 4. Conclusions and future work   

Identifying suitable cryo-EM search models is a key step in successful model fitting, especially for proteins which adopt different conformations. A key advantage of *MrBUMP* is that it automatically identifies and attempts to place a large number of potential cryo-EM search models. This ensures that if a low sequence-identity homologue exists in a similar conformation to the target protein, it will be found and fitted. This has been demonstrated in this study by the successful fitting of the PDB entry 5x9u-derived cryo-EM search model (27% sequence identity to the target) at several resolutions. The *MrBUMP* approach has proved popular in X-ray crystallography, where it removes the subjectivity of selecting the ‘best’ MR search model. Although the phases measured in cryo-EM allow one to see the target map, the same ambiguity can exist in choosing an atomic model for fitting, especially at lower resolutions.

There are several areas that we will focus on to improve the performance of *MrBUMP* in the future. One area that we will explore will be how to improve the quality of the cryo-EM search models that we identify. In crystallography, creating ensembles and truncating them based on the variation within the ensemble is a useful strategy for molecular replacement (Bibby *et al.*, 2012 ▸, 2013 ▸; Rigden *et al.*, 2018 ▸; Simpkin *et al.*, 2019 ▸; Keegan *et al.*, 2015 ▸; Leahy *et al.*, 1992 ▸; Adams *et al.*, 2010 ▸). In an unpublished study, we tested truncated cryo-EM search models with the GroEL data set. This strategy performed well for the high-resolution data sets (3.26–4 Å), but struggled at lower resolutions where the overall shape was more important. An alternative approach to deal with flexible regions might be to use a program such as *CONCOORD* (de Groot *et al.*, 1997 ▸) to generate a number of potential conformations for a given cryo-EM search model and trial these. Additionally, we can explore the use of sensitive sequence-searching software such as *HHpred* (Söding *et al.*, 2005 ▸; Zimmermann *et al.*, 2018 ▸) to identify more distantly related homologues and online databases of high-quality *de novo* model predictions such as those from the EBI and *AlphaFold*2 (Jumper *et al.*, 2021 ▸).

Here, we used *MOLREP* with the spherically averaged phased translation function (SAPTF) option selected. This is recommended for fitting small models into a larger map. However, where the cryo-EM search model constitutes a large part or the entire contents of the map, it may be better to use the phased translation function. Future research will explore this option in *MOLREP* as well as in other map-fitting programs.

Currently, *MrBUMP* uses the *MOLREP* score to assess the quality of the placed cryo-EM search models. We will further develop the scoring output to include *TEMPy* and other standard scores suitable for cryo-EM data.

In this research we found that segmenting the maps improved map fitting for higher resolution data sets (<8 Å), where the segmented maps were able to identify 22 additional solutions. Conversely, we found that map fitting performed better with the full maps for lower resolution data sets (≥8 Å), where the full maps were able to identify 17 additional solutions. We will therefore also explore the use of new segmentation methods as and when they are developed.

An added benefit of using segmented maps was a reduction in the run time of the program. *MrBUMP* (version 2.2.3) is currently available through the command line in *CCP*4, with plans to bring it to the *CCP-EM* GUI in the near future.

## Supplementary Material

Supplementary Figures and caption to Supplementary Table S1. DOI: 10.1107/S2059798321009165/qv5002sup1.pdf

Supplementary Table S1. DOI: 10.1107/S2059798321009165/qv5002sup2.xlsx

## Figures and Tables

**Figure 4:**
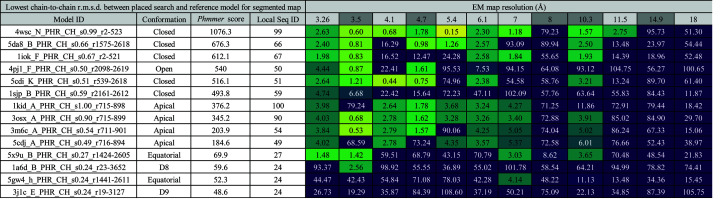
The lowest chain-to-chain r.m.s.d. between the placed cryo-EM search model and a reference model upon fitting a single copy into a segmented map. Columns and rows are as in Fig. 3 ▸.

**Table 1:** Information about GroEL data sets including the reported resolution, the *d*_99_ resolution calculated by *phenix.mtriage* (Afonine *et al.*, 2018 ▸), the symmetry imposed during reconstruction, the release year and the PDB code for deposited models where available The source organism is *E. coli* and the conformation is closed for all targets.

EMDB code	Resolution (Å)	*d*_99_ resolution (Å)	Imposed symmetry	Year released	PDB code for deposited model
EMD-3407	3.26	4.35	*C*7	2016	—
EMD-8750	3.5	3.5	*D*7	2017	5w0s
EMD-6422	4.1	4.18	*D*7	2015	—
EMD-5002	4.7	4.98	*C*7	2009	3c9v
EMD-1457	5.4	7.47	*D*7	2008	—
EMD-5338	6.1	4.78	*D*7	2011	—
EMD-1997	7	7.54	*C*7	2012	—
EMD-1998	8	8.96	*C*7	2012	4aaq
EMD-1042	10.3	10.2	*C*7	2003	1gr5
EMD-1080	11.5	12.86	*D*7	2004	—
EMD-1047	14.9	13.14	*C*7	2003	2c7e
EMD-5143	18	16.21	*C*1	2010	—
