# Supplementary material for: Redeployment of automated MrBUMP search-model identification for map fitting in cryo-EM
Source: Acta Crystallogr D Struct Biol. 2021 Oct 20;77(Pt 11):1378–85. doi: 10.1107/S2059798321009165 (PMC8561737; doi:10.1107/S2059798321009165)
Supplement: Supplementary file 1 [file d-77-01378-sup1.pdf]

STRUCTURAL
BIOLOGY

Volume 77 (2021)

Supporting information for article:

Redeployment of automated *MrBUMP* search model identification for map fitting in cryo-EM

Adam J. Simpkin, Martyn D. Winn, Daniel J. Rigden and Ronan M. Keegan

Table S1 Full results table for the GroEL case study. Information is provided on the resolution of the deposited data, the model from which the reference structure was generated, the type of map (full or segmented), information from the initial *MrBUMP* sequence search including phmmer score and local sequence identity, scores from *TEMPy* including global correlation and local correlation, the lowest chain-to-chain RMSD between the placed cryo-EM search model and a reference model, the number of molecules that were fitted within 5Å of the reference model and whether the solution appeared to be correct by visual inspection. Five columns (*TEMPy* global/local CC, lowest RMSD, number of molecules within 5Å and solution by eye) are colours on a scale from indigo to green to yellow, where indigo represents a poor score and yellow indicates a good score.

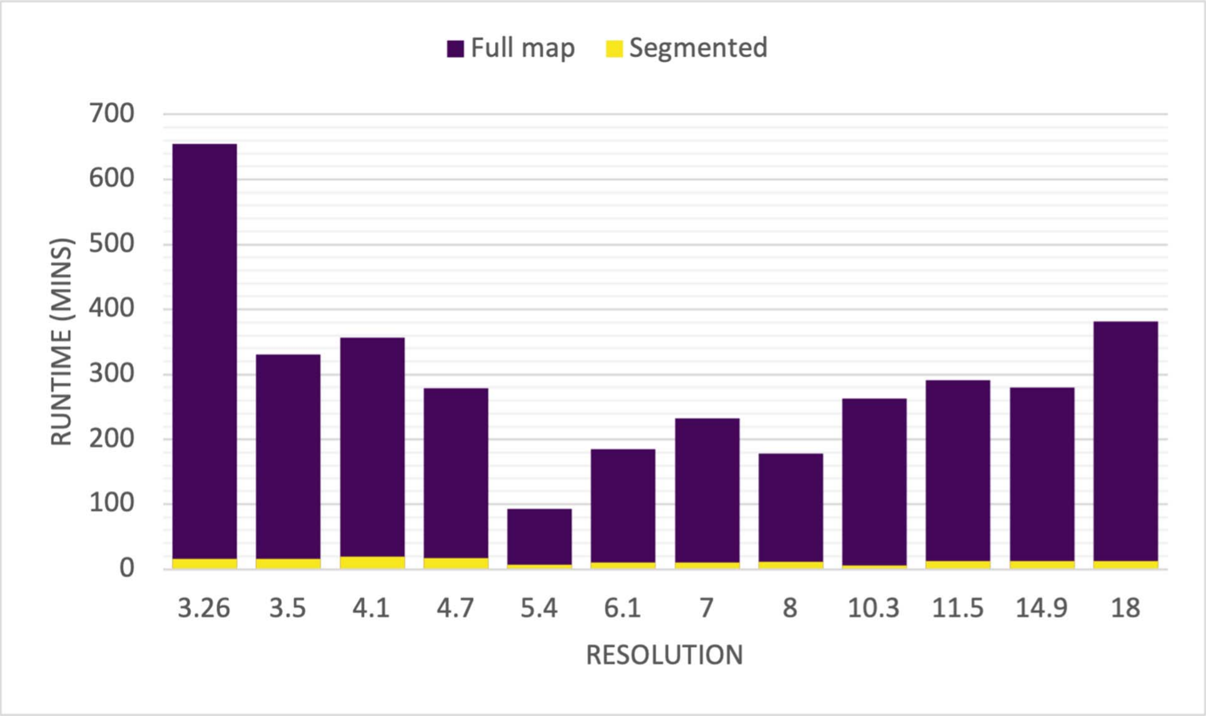

Figure S1 Timing comparisons between *MrBUMP* in the full maps (indigo) and in the segmented map (yellow).

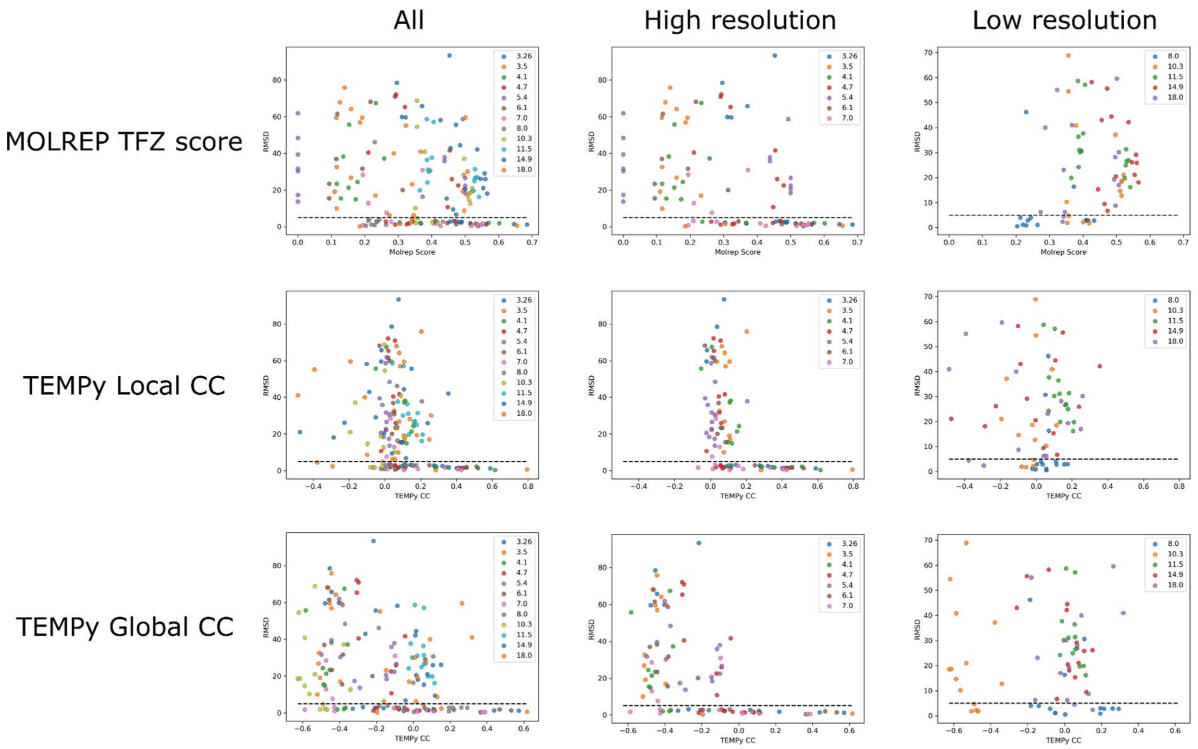

Figure S2 Comparison between different scoring metrics and RMSD from a reference structure for full maps. The columns show all data, high resolution data (<8Å) and low resolution data (≥8Å). The rows show *MOLREP* TFZ score, *TEMPy* Local CC score and *TEMPy* Global CC score.

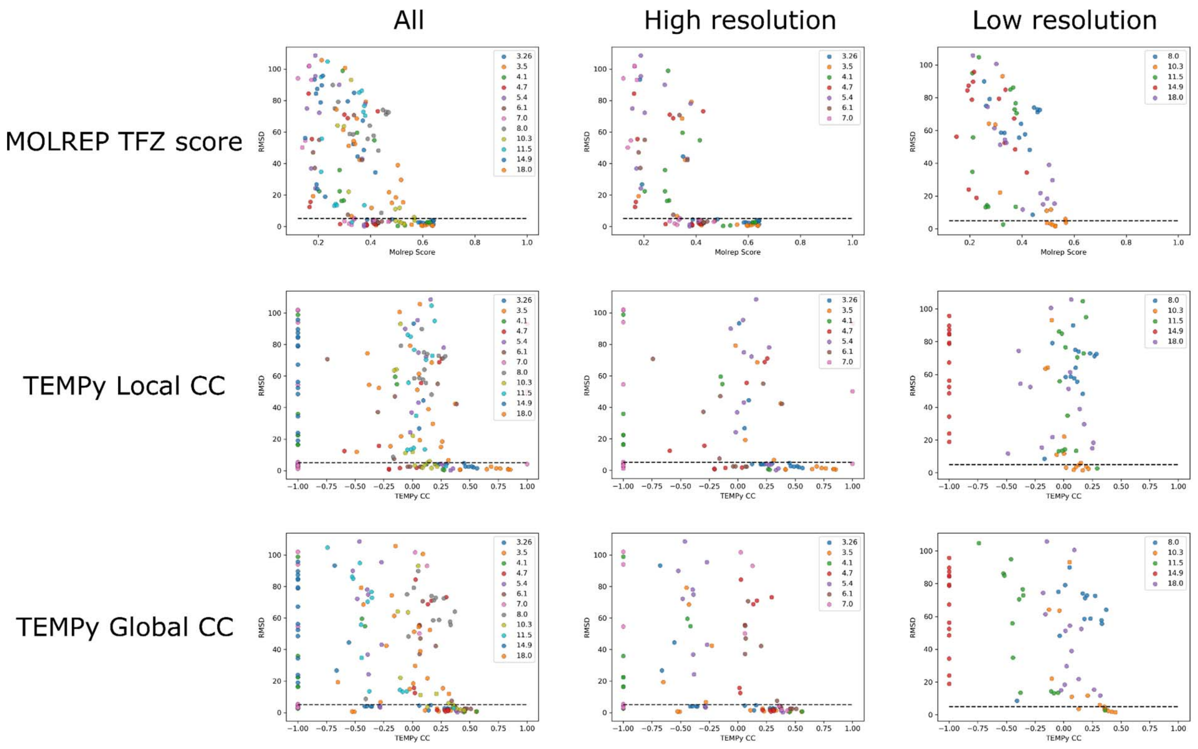

Figure S3 Comparison between different scoring metrics and RMSD from a reference structure for segmented maps. The columns show all data, high resolution data (<8Å) and low resolution data (≥8Å). The rows show *MOLREP* TFZ score, *TEMPy* Local CC score and *TEMPy* Global CC score.
